# Altered Levels of Natural Autoantibodies against Heat Shock Proteins in Pregnant Women with Hashimoto’s Thyroiditis

**DOI:** 10.3390/ijms25031423

**Published:** 2024-01-24

**Authors:** Diána Simon, Szabina Erdő-Bonyár, Katalin Böröcz, Noémi Balázs, Ahmed Badawy, Anna Bajnok, Jasper Nörenberg, Tímea Serény-Litvai, Ákos Várnagy, Kálmán Kovács, Eszter Hantosi, Emese Mezősi, Péter Németh, Tímea Berki

**Affiliations:** 1 Department of Immunology and Biotechnology, Clinical Center, Medical School, University of Pécs, 7624 Pécs, Hungary; 2 National Laboratory on Human Reproduction, University of Pécs, 7624 Pécs, Hungary; 3 Szentágothai Research Centre, University of Pécs, 7624 Pécs, Hungary; 4 Department of Obstetrics and Gynecology, Clinical Center, Medical School, University of Pécs, 7624 Pécs, Hungary; 5 First Department of Internal Medicine, Clinical Center, Medical School, University of Pécs, 7624 Pécs, Hungary

**Keywords:** natural autoantibodies, heat shock proteins, citrate synthase, pregnancy, Hashimoto’s thyroiditis

## Abstract

The function of natural autoantibodies (nAAbs) in maintaining immunological tolerance has been comprehensively explained; however, their function in pregnant patients dealing with autoimmune diseases has not been thoroughly investigated. As Hashimoto’s thyroiditis (HT) is the predominant organ-specific autoimmune condition of women of childbearing age, this study’s objective was to evaluate IgM and IgG nAAbs targeting mitochondrial citrate synthase (CS) and heat shock proteins (Hsp60 and Hsp70) in women diagnosed with HT who were pregnant (HTP). Serum samples collected from HTP and healthy pregnant (HP) women in the first and third trimesters were tested using in-house-developed enzyme-linked immunosorbent assays (ELISAs). Our findings indicate the stability of nAAbs against CS and Hsps throughout the pregnancies of both healthy women and those with HT. However, during both trimesters, HTP patients displayed elevated levels of IgM isotype nAAbs against Hsp60 and Hsp70 compared to HP women, suggesting a regulatory role of IgM nAAbs during the pregnancies of patients with HT. Nonetheless, levels of IgG isotype nAAbs against Hsps were lower solely in the third trimester among HTP patients, resulting in a higher IgM/IgG ratio, which indicates their importance in alterations of the nAAb network during pregnancy in patients with HT.

## 1. Introduction

Following the unveiling of natural autoimmunity, an increasing number of articles have emerged detailing the cells and molecules implicated. In relation to the concepts of ‘natural immunity’ and ‘natural autoimmunity’, it is crucial to emphasize a frequently observed misconception. In epidemiological literature, ‘natural immunity’ is commonly defined as a result of a wild-type infection, contrasting with vaccine-induced immunity [1,2]. Specifically, within the literature about the immune system, the term ‘natural autoimmunity’ denotes a complex network involving cells and their products, such as natural autoantibodies (nAAbs). This network act as a link between the innate and adaptive (specific) immune systems, as their cells express receptors and molecules characteristic of both systems [3,4,5,6,7,8]. Nevertheless, their functions in both normal and pathological conditions remain ambiguous.

The nAAbs constitute a network designed to safeguard an organism from internal and external dangers; however, they may also play a role in the development of autoimmune diseases [9]. Unlike the B2 B cell-derived antigen-specific antibodies produced by mature B cells through somatic hypermutation in a T-cell dependent pathway, nAAbs are produced by B1 B cells and marginal zone (MZ) B cells against evolutionarily conserved antigens without prior immunization [10]; they are polyreactive, having low-specificity and low-affinity towards antigens [9,11,12,13,14]. This network comprising nAAbs plays a crucial role in identifying and tagging invading microbes, thereby alerting initial responders of the innate immune system during an acute infection, and thus playing a crucial role in the initial stages of the immune response against pathogens. Their ability to bind both self-molecules and foreign molecules enables the binding of preserved self-molecules, including mitochondrial enzymes or heat shock proteins (Hsps). This mechanism conceals these molecules from the adaptive immune system, thus actively preventing the development of pathogenic autoimmunity [15]. They recognize and protect ancient epitopes of antigens, such as phospholipids, glycolipids, and glycoproteins [3,4,16], take part in the clearance of apoptotic cells and confer protection against potentially harmful substances like oxidized lipids and Hsps [17].

While natural IgM isotype antibodies serve a protective function, they also pose a potential risk of inducing inflammation through complement activation. If natural IgM antibodies enter tissues, particularly during endothelial damage, their presence triggers complement activation and subsequent tissue damage. This mechanism is frequently observed in autoimmune diseases [10]. Autoantibodies of the IgG isotype are prevalent in human serum, yet their levels may undergo dynamic changes depending on various factors, such as age, gender, and the impact of illnesses and vaccinations [18,19,20,21,22]. In our previous studies, we investigated the nAAb profile targeting a conserved mitochondrial enzyme called citrate synthase (CS) among both healthy individuals and patients diagnosed with systemic autoimmune diseases. Research has revealed that IgM nAAb levels directed against CS tend to be more individual-specific and consistent, while IgG levels may fluctuate under specific conditions, implying that these nAAbs may undergo adaptive changes upon a competent immunogenic trigger [19,20,21,22,23]. We also investigated nAAbs against Hsps in healthy individuals post-vaccination for SARS-CoV-2. Our findings indicated a correlation between the levels of anti-Hsp70 nAAbs and vaccine effectiveness [21]. Anti-Hsp70 nAAbs have been described as potential important regulators of establishing the proper amount of their target antigen necessary for the maintenance of fetal tolerance during normal pregnancy [24]. Furthermore, it has been suggested that during pregnancy nAAbs may be involved in controlling inflammation that could compromise a pregnancy [25].

In the present study, our aim was to compare the levels of IgG and IgM nAAbs in pregnant women, distinguishing between those who were healthy and those with autoimmune conditions. We also aimed to monitor alterations in these levels during the first and third trimesters. As Hashimoto’s thyroiditis (HT) is the most frequent organ-specific autoimmune condition among women of reproductive age, we conducted the investigation using women with HT. Additionally, women positive for anti-thyroid autoantibodies face a fourfold increased risk of miscarriage and a twofold heightened risk of preterm delivery [26]. We hypothesized that in pregnant women who had HT (HTP), this phenomenon could be attributed to the disruption of immunological tolerance towards the fetus, in which the network of nAAbs may play a significant role. We are confident that the findings herein described can offer novel insights into the role of nAAbs in facilitating tolerance induction during pregnancy.

## 2. Results

### 2.1. Increased IgM and Decreased IgG Levels of Anti-Hsp60 and Hsp70 Autoantibodies in HTP 

**Figure 1:**
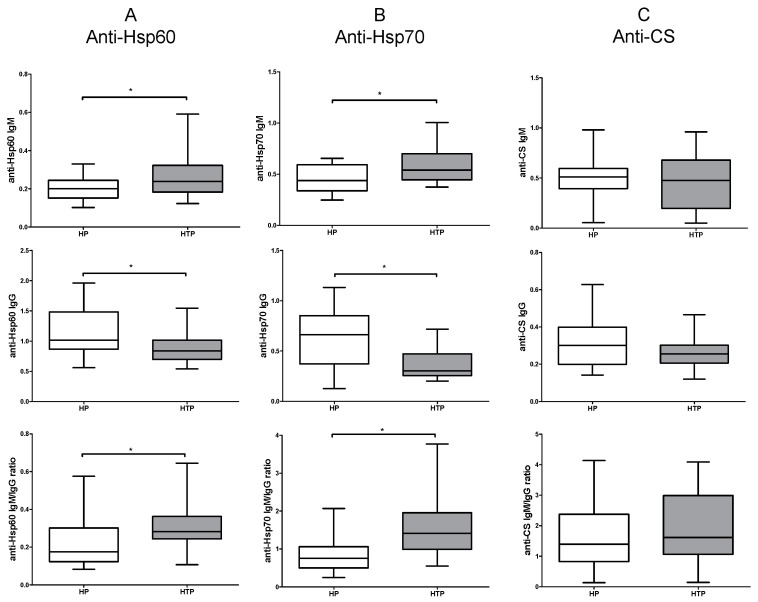
Levels of natural autoantibodies (nAAbs) in healthy (HP) and Hashimoto’s thyroiditis pregnant (HTP) women. Levels of anti-heat shock protein (Hsp) 60 IgM and IgG, and the IgM/IgG ratio (**A**); levels of anti-Hsp70 IgM and IgG, and the IgM/IgG ratio (**B**); and levels of anti-citrate synthase (CS) IgM and IgG, and the IgM/IgG ratio (**C**). Boxes show interquartile ranges (IQRs), horizontal lines represent medians, and whiskers indicate the lowest and highest values. n_(HP)_ = 14, n_(HTP)_ = 14 * *p* < 0.05.

Initially, we investigated the levels of IgM and IgG nAAbs targeting CS, Hsp60, and Hsp70 in both healthy pregnant (HP) and HTP groups. Women with HTP exhibited increased levels of IgM autoantibodies against Hsp60 and Hsp70 in comparison to the HP group. Contrastingly, diminished levels of IgG autoantibodies against Hsp60 and Hsp70 were noted in the HTP group as opposed to the HP group (Figure 1A,B). There were no discernible differences in the levels of anti-CS IgM and IgG autoantibodies between HP and HTP groups (Figure 1C). Moreover, we assessed the ratio of IgM to IgG natural autoantibodies: an elevated IgM/IgG ratio was detectable in women with HTP compared to those in the HP group for autoantibodies against Hsp60 and Hsp70 (Figure 1A,B). No noticeable difference was found in the ratio of IgM to IgG autoantibodies against CS between HP and HTP groups (Figure 1C).

### 2.2. Altered Levels of Both IgM and IgG nAAbs against Hsp60 and Hsp70 in the Third Trimester of HTP Women Resulting in Their Elevated Ratios

**Figure 2:**
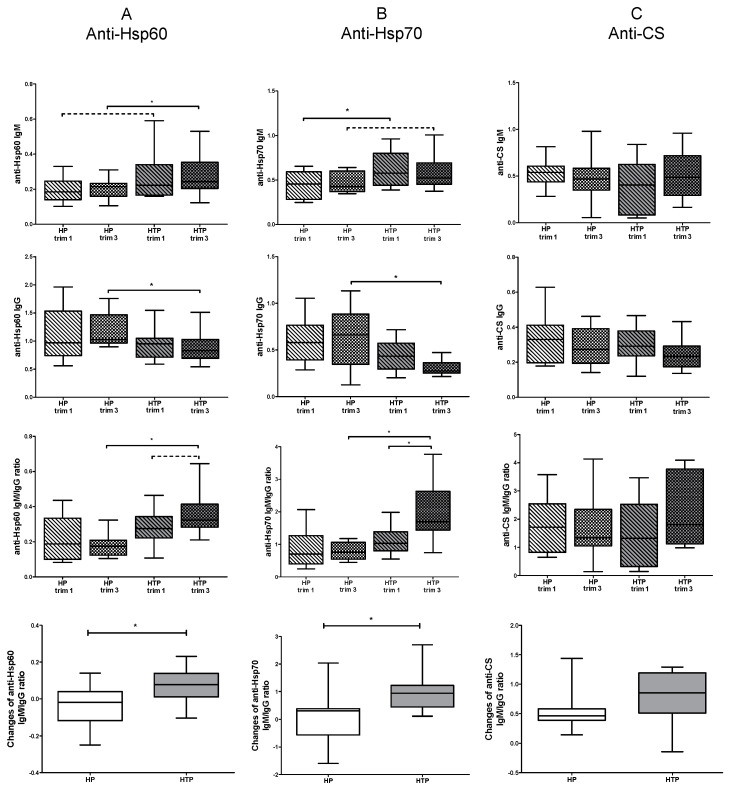
Levels of nAAbs in the first and third trimesters of healthy (HP) and Hashimoto’s thyroiditis pregnant (HTP) women. Levels of anti-Hsp60 IgM and IgG, IgM/IgG ratios, and changes in IgM/IgG ratios (**A**); levels of anti-Hsp70 IgM and IgG, IgM/IgG ratios, and changes in IgM/IgG ratios (**B**); and levels of anti-CS IgM and IgG, IgM/IgG ratios, and changes in IgM/IgG ratios (**C**). Boxes show interquartile ranges (IQRs), horizontal lines represent medians, and whiskers indicate the lowest and highest values. n_(HP)_ = 14, n_(HTP)_ = 14 * *p* < 0.05.

Subsequently, our interest turned to identifying the trimester during pregnancy in which the previously noted differences manifested. To investigate this, we separately examined samples from the first trimester and third trimester. During the third trimester, there was a significant increase in levels of anti-Hsp60 IgM autoantibodies, and a tendency for an increase was observed in the first trimester of individuals with HTP compared to the corresponding trimester in the HP group (Figure 2A). Significantly higher levels of anti-Hsp70 IgM autoantibodies were observed in the first trimester of women with HTP, and there was a tendency for elevation in the third trimester compared to the corresponding trimester in the HP group (Figure 2B).

There was a significant decrease in anti-Hsp60 and anti-Hsp70 IgG autoantibodies during the third trimester of women with HTP compared to the third trimester of HP women. Nevertheless, no significant differences were observed in the levels of IgM and IgG nAAbs against Hsps between the first and third trimesters in either the HTP group or the HP group (Figure 2A,B). Upon examination of the trimesters, no differences were noted in levels of anti-CS autoantibodies between HP and HTP groups (Figure 2C).

When analyzing IgM/IgG ratios in the first and third trimesters, we observed that the IgM/IgG ratios of anti-Hsp60 and anti-Hsp70 antibodies were both higher in the third trimester of HTP women compared to the third trimester of HP women. Additionally, the IgM/IgG ratio was significantly higher in the third trimester compared to the first trimester of women with HTP for anti-Hsp70 autoantibodies, and there was a tendency for an increase in anti-Hsp60 autoantibodies.

In our final analysis, we investigated alterations in the IgM/IgG ratio of measured nAAbs between the first and third trimesters. We observed significantly greater changes in the IgM to IgG ratio between trimesters for anti-Hsp60 and anti-Hsp70 autoantibodies in women with HTP compared to those in the HP group (Figure 2A,B). No differences were identified between HP and HTP groups when assessing the IgM/IgG ratio of autoantibodies against CS (Figure 2C).

## 3. Discussion

The nAAb network plays an important regulatory role in the maintenance of immunological tolerance. It is capable of inhibiting inflammatory processes and counteracting the advance of autoreactive immune reactions. The literature and our own observations support the idea that natural IgM autoantibody levels are more stable. However, B cells undergoing isotype switching respond to activation by regulatory IgG nAAb production [17,19,20,21,22,23]. A microarray study of 166 healthy adults showed that a large number of IgG isotype nAAbs are commonly found in all human sera [18]. These IgG isotype nAAbs react with antigens from human tissues and organs, and their diversity is strongly influenced by the age, sex, and disease of an individual.

In addition to protecting self-structures, the nAAb network has also been hypothesized to have a role in the creation and maintenance of fetal tolerance [25,27]. Thus, we extended our study to the determination of IgM and IgG nAAbs in serum samples from HTP and HP women. We assessed nAAbs directed against the highly-conserved CS and Hsps. Hsps are crucial elements in stress response and apoptosis regulation. CS, a mitochondria-encapsulated protein, is not released during apoptotic processes in fetal development. Given that nAAbs have been identified as potential important regulators of the available amount of their target antigens, it is reasonable to hypothesize that any lack of change in the target antigen’s quantity would be accompanied by an absence of alteration in the magnitude of the autoantibody [24]. Therefore, no change in the level of anti-CS nAAbs could be anticipated during pregnancy, which was confirmed by our findings showing that the levels of IgM and IgG nAAbs against CS and their ratios were similar and stable during the pregnancies of both HTP and HP women.

Extracellular Hsp70 can represent a danger signal and elicit proinflammatory immune responses [28]. Additionally, Hsp70 was proven to be able to activate the complement system via the classical pathway [29]. On the other hand, extracellular Hsp70 was reported to be cytoprotective and capable of cell protection from apoptosis [30]. Among Hsps, Hsp70 has been the most studied during pregnancy. The exact role of Hsp70 at the maternal–fetal interface is currently unclear, but its importance in blastocyst development and implantation, and as a signaling system for apoptotic processes during fetal development, has been speculated [31]. Li et al. [32] described no elevation in the expression of Hsp70 by the placenta as pregnancy processed. Fukushima et al. [33] compared concentrations of Hsp70 in the serum during three trimesters and did not find significant differences; thus, they considered its amount in the serum to be stable throughout pregnancy. According to Molvarec et al., anti-Hsp70 nAAbs could be one of the key regulatory elements in accomplishing the appropriate level of circulating Hsp70 needed for the maintenance of immune tolerance during fetal development in normal pregnancy [24], which is supported by our results, which show no difference in levels of IgM and IgG autoantibodies against Hsps in the serum of HP women between the first and third trimesters. However, we found higher anti-Hsp70 and anti-Hsp60 IgM nAAb levels in the first trimester of HTP women, which remained elevated throughout the whole pregnancy, suggesting the presence of higher Hsp60 and Hsp70 levels in HTP women compared with those of HP women. Interestingly, higher levels of Hsp70 were observed in patients with preeclampsia, hemolysis, elevated liver enzymes, low platelet count (HELLP) syndrome, gestational diabetes, and preterm delivery [34,35,36,37]. Nonetheless, we measured lower anti-Hsp70 and Hsp60 IgG nAAb levels in the third trimester of HTP women compared with those of HP women, resulting in a prominent difference in the IgM/IgG ratios of HTP and HP women. Furthermore, changes observed in IgM/IgG ratios of autoantibodies against Hsp60 and Hsp70 between the first and third trimesters were larger in HTP women than they were in HP women; thus, the elevated Ig/IgG ratios of anti-Hsp nAAbs in the third trimester may be important components of the regulatory function exerted by the nAAb network during pregnancy in HT women.

Our findings reveal alterations in the nAAb networks of HTP women when compared to those of HP women. These results provide additional support to our hypothesis that this network can play a pivotal role in establishing and maintaining immunological tolerance toward the fetus, and that it can be influenced by autoimmune processes. Results herein presented are initial findings from an ongoing, more extensive study of the constituents of immunological tolerance during pregnancy and its common complications focusing on the possible roles of the nAAb network, which may help identify potential therapeutic targets.

## 4. Materials and Methods

### 4.1. Selected Individuals

For our research, we recruited fourteen pregnant women with HT (HTP) and fourteen healthy pregnant (HP) women, all belonging to the Caucasian population. As part of the enrollment process, we thoroughly reviewed the medical history of each participant to screen for autoimmune and genetic diseases. Additionally, during the collection of peripheral blood samples, routine laboratory parameters were examined to screen for current infections and diseases. Pregnancies were monitored throughout their course.

Selected HTP participants were euthyroid, anti-thyroid antibody positive, and aged 20–40 years. Inclusion criteria for HTP participants included TSH levels < 4.2 mU/L, positivity for at least one anti-thyroid autoantibody, a normal menstrual cycle before conception, a normal pregnancy, and regular attendance at prenatal care. Exclusion criteria comprised a history of other autoimmune diseases, chronic diseases, genetic abnormalities, significant obesity (BMI > 35 kg/m^2^), a history of hypothyroidism, twin pregnancy, gestational diabetes, preeclampsia, toxemia, smoking, fetal abnormalities detected during ultrasound screening, and preterm birth.

Age-matched HP women were chosen as controls. Inclusion criteria for HP women included an age of 20–40 years, a normal menstrual cycle before conception, a normal pregnancy, and regular attendance at prenatal care. Exclusion criteria encompassed elevated TSH (>4.2 mU/L), positivity for anti-thyroid antibodies, a history of other autoimmune diseases, chronic diseases, genetic abnormalities, significant obesity (BMI > 35 kg/m^2^), twin pregnancy, gestational diabetes, preeclampsia, toxemia, smoking, fetal abnormalities detected during ultrasound screening, and preterm birth.

Peripheral blood samples were collected from all pregnant women during the first trimester (13–14 weeks) and third trimester (33–34 weeks) of pregnancy. Following approval from the Regional Research Ethics Committee of the University of Pécs Medical Centre (RIKEB 5913/2015), written informed consent was obtained from all participants for their involvement in this study. The period of patient recruitment, sample collection, and processing spanned from March 2019 to March 2022. As samples were collected from all pregnant women in both the first and third trimesters, the sample collection period extended over a considerable duration. The recruitment process began by selecting participants for the HTP group. In parallel, a non-random procedure was used to select participants of similar ages for the HP group.

### 4.2. Measurement of nAAbs against CS

Levels of anti-citrate synthase (anti-CS) IgM and IgG autoantibodies were determined using an in-house enzyme-linked immunosorbent assay (ELISA), as previously described [21]; ninety-six-well polystyrene plates (Nunc, Roskilde, Denmark) were coated with citrate synthase from porcine heart (Sigma-Merck, Munich, Germany) in a 0.1 M bicarbonate buffer, pH 9.6, at 4–8 °C overnight [19]. Following the saturation of non-specific binding sites with a solution of 0.5% polyvinyl alcohol and bovine gelatin in a 2:1 ratio, serum samples were incubated in duplicate at 1:100 dilution in a washing buffer (PBS, 0.05% Tween 20) for 50 min at 37 °C. Finally, after extensive washing, the plate was incubated with horseradish peroxidase (HRP)-conjugated anti-human IgG- and IgM-specific secondary antibodies (Agilent-Dako Santa Clara, CA, USA) for 45 min at 37 °C. The reaction was developed using 3,3′,5,5′-Tetramethylbenzidine (TMB) (Sigma-Merck, Munich, Germany), and optical density (OD) was measured at λ = 450/620 nm using the BEP 2000 Advance automated system (Siemens, Marburg, Germany).

### 4.3. Measurement of nAAbs against Hsp60 and Hsp70

An in-house ELISA was used to determine anti-Hsp60 and anti-Hsp70 IgM and IgG autoantibodies according to the protocol described previously [21]. Briefly, ninety-six-well polystyrene plates (Nunc, Roskilde, Denmark) were coated with human Hsp60 (Abcam, Waltham, Boston, MA, USA) and human Hsp70 (Abcam, Waltham, Boston, MA, USA) at a concentration of 1 μg/mL in ELISA coating buffer (Bio-Rad, Hercules, CA, USA) at 4 °C overnight. After blocking the wells with a 2:1 solution of 0.5% polyvinyl alcohol and bovine gelatin, serum samples were incubated in duplicate at 1:200 dilution in washing buffer (PBS, 0.05% Tween 20) for 1 h at 37 °C. After incubation with HRP-conjugated anti-human IgG- and IgM-specific secondary antibodies (Agilent-Dako Santa Clara, CA, USA) for 40 min at 37 °C, the reaction was visualized using TMB solution, and the OD was read at λ = 450/620 nm using the BEP 2000 Advance automated system (Siemens, Marburg, Germany).

### 4.4. Statistical Analysis

Statistical evaluation was performed using the SPSS version 27.0 statistics package (IBM, Armonk, NY, USA). Mann–Whitney U tests and Wilcoxon signed-rank were used when *p* values < 0.05 were considered significant. To determine the power of our experiments, we performed a post hoc power analysis. For a tested sample size and α = 0.05 significance level, the β values of the significant differences between HP and HTP groups were 0.212 for anti-Hsp60 IgG, 0.171 for anti-Hsp60 IgM, 0.033 for anti-Hsp70 IgG, and 0.252 for anti-Hsp70 IgM autoantibodies, while the β values of non-significant differences were 0.702 for anti-CS IgG and 0.898 for anti-CS IgM autoantibodies. The calculated power (1-β) values for significant differences between HP and HTP groups were 78.8% for anti-Hsp60 IgG, 82.9% for anti-Hsp60 IgM, 96.7% for anti-Hsp70 IgG, and 74.8% for anti-Hsp70 IgM autoantibodies, while values obtained for non-significant differences (29.8% for anti-CS IgG and 10.2% for anti-CS IgM autoantibodies) were much lower, as expected [38,39].

## Data Availability

Data are contained within the article.
